# Caregiver Health and Well-Being, and Financial Strain

**DOI:** 10.1093/geroni/igaa057.2374

**Published:** 2020-12-16

**Authors:** Susan Reinhard, Lynn Feinberg

**Affiliations:** 1 AARP, Washington, District of Columbia, United States; 2 AARP Public Policy Institute, Washington, District of Columbia, United States

## Abstract

Family caregivers often face key challenges when caring for a relative or close friend with health or functional needs. This paper presents findings from Caregiving in the U.S. 2020 on the impact of caregiving on the physical health and well-being of family caregivers and the financial impacts of family care. The data suggest that the caregiver’s own health has declined, with 1 in 5 (21%) saying they are in fair to poor health themselves, up from 17 percent in 2015. Nearly 1 in 4 (23%) feel caregiving has made their health worse. Nearly 4 in 10 (38%) family caregivers of adults experience a moderate to a high degree of financial strain from providing care. Forty-five percent have experienced at least one financial impact (e.g., stopped savings, debt, ability to pay bills, and ability to afford necessary expenses, like food). These findings reveal that because family caregiving today is more complex, costly, and stressful than in the past, action is needed to recognize and support family caregivers in the U.S adequately.

